# Assessment of nutritional status and quality of life among hemodialysis patients in Aswan

**DOI:** 10.1186/s12882-025-04555-9

**Published:** 2025-12-01

**Authors:** Aml Ahmed Sayed, Hanaa Mohammed Eid El sayed, Marwa Ahmed Abdelhameid

**Affiliations:** 1 Internal Medicine Department, Faculty of Medicine, Aswan University, Aswan, Egypt; 2 Internal Medicine Department, Faculty of Medicine for Girls, Al-Azhar University, Cairo, Egypt; 3 Department of Internal Medicine, Faculty of Medicine, Aswan University, Aswan, 81528 Egypt

**Keywords:** Nutritional status, Quality of life, Hemodialysis

## Abstract

**Background:**

Malnutrition is prevalent in patients with end-stage kidney disease undergoing hemodialysis (HD). It adversely affects the prognosis and is linked to elevated morbidities, mortalities as well as diminished quality of life (QOL). This study attempted to assess the nutritional status and QOL among HD cases.

**Patients and methods:**

This cross-sectional study was performed at Aswan University Hospital (Aswan, Egypt) in the interval from December 2022 until December 2023. The study included 150 patients on regular HD. All patients underwent comprehensive examinations, including a complete medical history of age, sex, body mass index (BMI), marital status, occupation, education, duration of dialysis, number of sessions per week. Investigations included complete blood count, urea, creatinine, total iron binding capacity, ferritin, albumin, cholesterol and triglyceride. The nutritional state was evaluated using the Subjective Global Assessment score (SGA) and QOL was assessed utilizing the 12-item Short Form Survey (SF-12).

**Results:**

The mean ± SD of patient’s age was 48.69 ± 14.04 years. Subjects comprised 70 (46.67%) males and 80 (53.33%) females. The mean ± SD of their BMI was 22.05 ± 5.27 kg/m^2^. The prevalence of malnutrition was 86%; 105 (70%) patients exhibited mild to moderate malnutrition. The current study revealed a marked negative association between SGA score and age (*P* = 0.029), BMI (*P* = 0.002), TIBC (*P* < 0.001), ferritin (*P* < 0.001), TG (*P* = 0.029), cholesterol (*P* < 0.001) and albumin (*P* < 0.001). Furthermore, there was a substantial positive correlation between the SGA score and duration of dialysis (*P* < 0.001). Regarding QOL, there was a marked negative association between the physical component summary (PCS) and age (***P* < 0.001**), SGA score (***P* = 0.042**) and BMI (***P* = 0.024**).

**Conclusion:**

Malnutrition is prevalent among hemodialysis patients in Aswan, particularly in individuals receiving long-term hemodialysis. The majority of patients exhibited mild to moderate malnutrition, whereas severe malnutrition was infrequent.

**Clinical trial number:**

Not applicable.

## Introduction

Chronic kidney disease (CKD) is characterized by the presence of an estimated glomerular filtration rate (e GFR) < 60 mL/min/1.73 m² of body-surface area or a urinary albumin excretion of >30 mg/24 hr or both for more than three months [1]. End stage renal disease (ESRD) represents the final and permanent phase of chronic kidney disease, individuals with ESRD necessitate either dialysis or kidney transplantation for survival [2].

CKD represents an increasing global health burden. The 2017 Global Burden of Disease study reported 697.5 million CKD cases, indicating a global prevalence of 9.1%. About 4 million individuals necessitate renal replacement therapy [3]. Approximately 13% of the adult Egyptian population is affected by CKD, leading to significant morbidity, mortality and health care costs [4].

Protein-energy wasting (PEW) occurs frequently in patients receiving maintenance hemodialysis (MHD) with a worldwide prevalence that varies between 28% and 54% [5]. Numerous contributing factors lead to the onset of PEW in MHD cases, including nutrient and amino acid depletion, elevated energy expenditure, dialysis-induced muscle catabolism, anabolic hormone resistance, inadequate metabolic acidosis correction, poor appetite, inadequate dialysis, suboptimal dietary intake, taste alterations, insulin resistance, diminished functional capacity, and psychological factors such as depression and lack of social support [6].

The National Kidney Foundation’s Kidney Disease Outcome and Quality Initiative (KDOQI) define SGA as the recommended clinical nutrition assessment tool with prognostic relevance for individuals with CKD [7].

Malnutrition is associated with elevated cardiovascular disease risks, increased mortalities, morbidities and hospitalization, resulting in poor outcomes. It negatively impacts patient’s QOL, including emotional, physical and psychosocial health [8]. Several factors, including anemia, age, duration of hemodialysis, number of comorbidities and number of medications are associated with the QOL of hemodialysis cases. HD can improve patient’s QOL by treating symptoms like fluid retention, loss of appetite, nausea and vomiting. People on dialysis can continue working, traveling and engaging in other preferred activities which can improve their QOL [9].

## Patients and methods

### Study design, setting and patients

This cross-sectional study included 150 individuals from the dialysis unit at Aswan University Hospital (**Middle Eastern community in Egypt)**, conducted between December 2022 and December 2023. Two hundred patients were undergoing MHD at Aswan University Hospital’s hemodialysis unit. We recruited 150 patients, excluding 50, which included ten patients < 18 years of age and 40 individuals on HD for less than six months. All Patients above 18 years on MHD for more than six months were included in the study, while infectious, chronic inflammatory diseases, pregnancy, malignancy and other debilitating diseases were excluded.

### Data collection

Data collected from eligible patients included full medical history; name, sex, body mass index (BMI), age, marital status, education, occupation, duration of dialysis, number of sessions per week, history of smoking, diabetes, hypertension, stroke, cardiac or peripheral vascular disease(PVD). Additionally, the assessment included complete blood count (CBC), urea, creatinine, total iron binding capacity (TIBC), ferritin, albumin, cholesterol and triglycerides (TG). Nutritional state was evaluated utilizing the **SGA score**, whereas QOL was assessed according to the **12-item Short Form Survey (SF-12)**, the questionnaires had been implemented at the dialysis unit with the help of study coordinator.

The **SGA** is an extensive approach for evaluating malnutrition in multiple diseases including HD. This approach is cost-efficient and effective for assessing individuals’ nutritional status. This method necessitates no laboratory data and is acknowledged as a reliable tool for evaluating nutritional status in individuals with HD. The questionnaire comprises 5 components of a medical history (weight change, dietary intake, gastrointestinal symptoms, functional capacity, disease and its relation to nutritional requirements) and 3 components of a brief physical examination (signs of fat and muscle wasting, nutrition-associated alternations in fluid balance). We assessed subcutaneous fat loss, the occurrence of ankle and sacral edema and muscle wasting during the physical examination. Each feature was individually assessed and categorized as A, B or C to indicate malnutrition extent. Subsequently, the SGA ratings were converted to numerical equivalents: a score up to 10 indicates well-nourished status, scores from 10 to 17 reflect mild to moderate malnutrition while scores exceeding 17 denote severe malnutrition Table 1 [10].

**Table 1:**
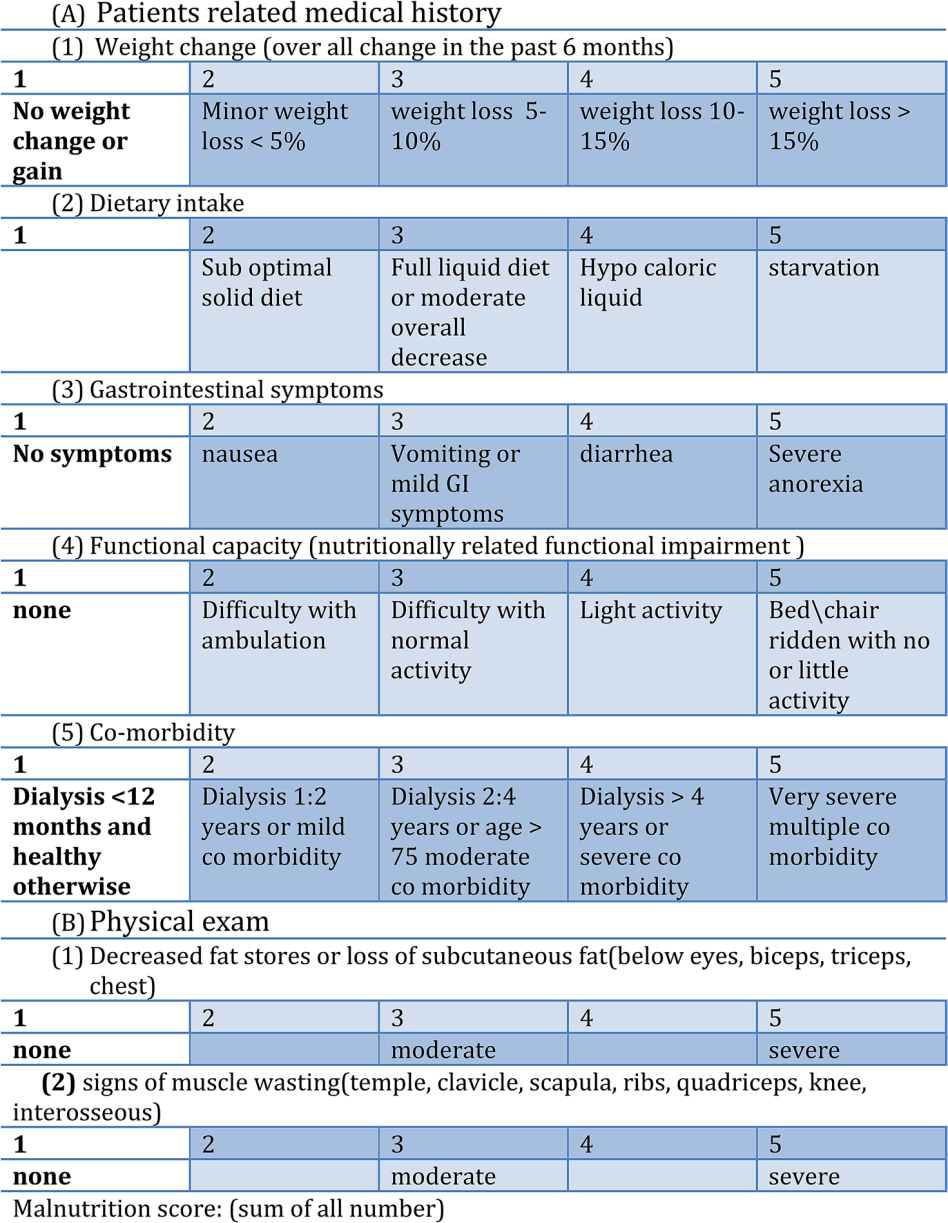
SGA score

**SF-12 serves** as a concise alternative to the SF-36 and is widely utilized in health outcomes surveys. It comprises 12 items that represent eight health domains designed to generate two summary scores: the mental component summary (MCS-12) as well as the physical component summary (PCS-12) [11]. Each score is derived from six items which include two to six response categories, the scoring yields two summary measures: the PCS and MCS: utilize the norm-based scoring system to interpret PCS and MCS scores, with a mean of 50 and a standard deviation of 10 in the general population, Scores above 50 indicate a better-than-average health-related quality of life, while scores below 50 suggest below-average health [12] Table 2.

**Table 2:** The 12-item short form health survey (SF-12)

Scales	No	Items	Response categories
PCS-12	**1**	General health	Excellent/Very good/Good/Fair/Poor
	**2**	Moderate activities	Limited a lot/Limited a little/Not limited at All
	**3**	Climb several flights of stairs	Limited a lot/Limited a little/Not limited at All
	**4**	Accomplished less (physical)	Yes/No
	**5**	Limited in kind of work	Yes/No
	**8**	Pain – interference	Not at all/A little bit/Moderately/Quite a bit/Extremely
MCS-12	**6**	Accomplished less (emotional)	Yes/No
	**7**	Did work less careful	Yes/No
	**9**	Calm and peaceful	All of the time/Most of the time/A good bit of the time/Some of the time/A little of the time/None of the time
	**10**	Energy	All of the time/Most of the time/A good bit of the time/Some of the time/A little of the time/None of the time
	**11**	Downhearted and blue	All of the time/Most of the time/A good bit of the time/Some of the time/A little of the time/None of the time
	**12**	Social limitations – time	All of the time/Most of the time/ Some of the time/A little of the time/None of the time

### Study outcomes

The study’s primary outcome was the prevalence of malnutrition among HD patients. The secondary outcomes included the association between nutritional status, QOL and other variables.

### Ethical statement

The study complies with both national and international ethical guidelines. There are no potential hazards for research participants in terms of economic, legal, physical, social, psychological or any other variables. The study’s risks, objectives and advantages were detailed to participants. The participants in the study provided written informed consent. The study adhered to the Declaration of Helsinki for human subjects. Additionally, the ethics committee of the Faculty of Medicine at Aswan University, Egypt, reviewed and approved the study (Asw.uni./523/3/21).

### Statistical analysis

SPSS v26 (IBM Inc., Armonk, NY, USA) was used for statistical analysis. We employed the Shapiro-Wilks test and histograms to assess data distribution normality. A post-hoc test (Tukey) and ANOVA (F) tests analyzed quantitative parametric data, which were presented as mean and standard deviation (SD). Quantitative non-parametric data analysis was conducted utilizing the Kruskal -Wallis test and presented as the median and inter quartile range (IQR). Group comparisons consisted of the Mann-Whitney test. The Chi-square test was employed to analyze qualitative variables which were presented as frequency and percentage (%). We conducted Pearson’s correlation to evaluate the correlation between two quantitative parametric variables. A two-tailed P value ≤ 0.05 was considered statistically significant.

## Results

The present study comprised 150 subjects from Aswan University Hospital. Patient’s mean age was 48.69 ± 14.04 years. There were 70 (46.67%) males and 80 (53.33%) females. The mean ± SD of their BMI was 22.05 ± 5.27 kg/m^2^.The mean duration of dialysis was 2:6 years. The main causes of ESRD were hypertension (40%), followed by diabetes (18%), glomerulonephritis (15%), idiopathic etiology (11%), obstructive uropathy (8%), lupus nephritis (4%) and polycystic kidney disease (4%).

Only 3 (2%) patients received four sessions per week, 113 (75.33%) patients received three sessions per week, 33 (22%) patients received two sessions per week and 1 (0.67%) patient received one session per week. We found that 19 (12.67%) subjects were single, 113 (75.33%) were married, 12 (8%) were divorced and 6 (4%) were widows. Only 31 (20.67%) patients are employed. Furthermore, 101 (67.33%) patients are educated. Among them, 23 (22.77%) have completed low-level education, 53 (52.48%) have completed moderate-level education and 25 (24.75%) have completed high-level education.

**Fig. 1:**
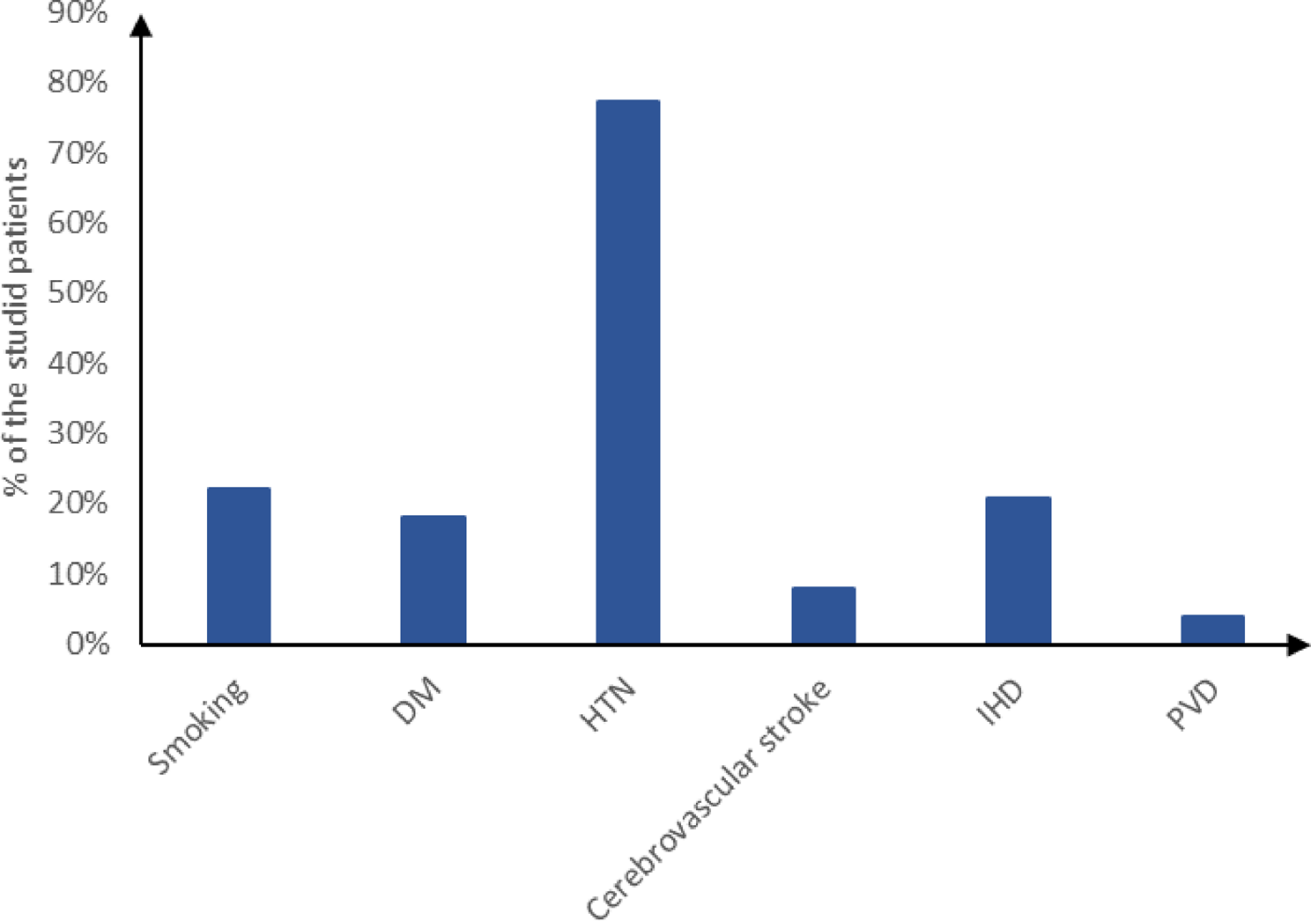
Comorbidities of the studied patients. DM: diabetes mellitus, HTN: Hypertension, IHD: ischemic heart disease, PVD: peripheral vascular diseases

As shown in Figure 1, there were 33 (22%) Smokers, 27 (18%) diabetic patients, 116 (77.33%) hypertensive patients, 12 (8%) patients with Cerebrovascular stroke, 31 (20.67%) patients with ischemic heart disease (IHD) and 6 (4%) patients with PVD.

Table 3 presents the laboratory data of the examined patients. The results indicated that 134 patients exhibited anemia: 45 (33.58%) had mild anemia (Hb < 11.5 g\dL), 82 (61.19%) had moderate anemia (Hb < 10 g\dL), and 7 (5.22%) had severe anemia (Hb < 7 g\dL).

**Table 3:** Laboratory investigations of the studied patients

		*n* = 150
Hb (g/dL)		9.55 ± 1.57
Anemia (*n* = 134)	**Mild**	45 (33.58%)
	**Moderate**	82 (61.19%)
	**Severe**	7 (5.22%)
WBCs (x10^9^/L)		6.58 ± 2.18
Platelets (x10^9^/L)		229.39 ± 73.59
Creatinine (mg/dL)		10.05 ± 2.15
Urea (mg/dL)		110.99 ± 29.17
TIBC (µg/dL)		246.5 ± 82.33
Ferritin (ng/mL)		157 (79–226)
Cholesterol (mg/dL)		172.47 ± 48.8
TG (mg/dL)		139 (90–200)
Albumin (g/dL)		3.78 ± 0.69

Hb: hemoglobin, WBCs: white blood cells, TIBC: total iron binding capacity, TG: triglycerides

**Fig. 2:**
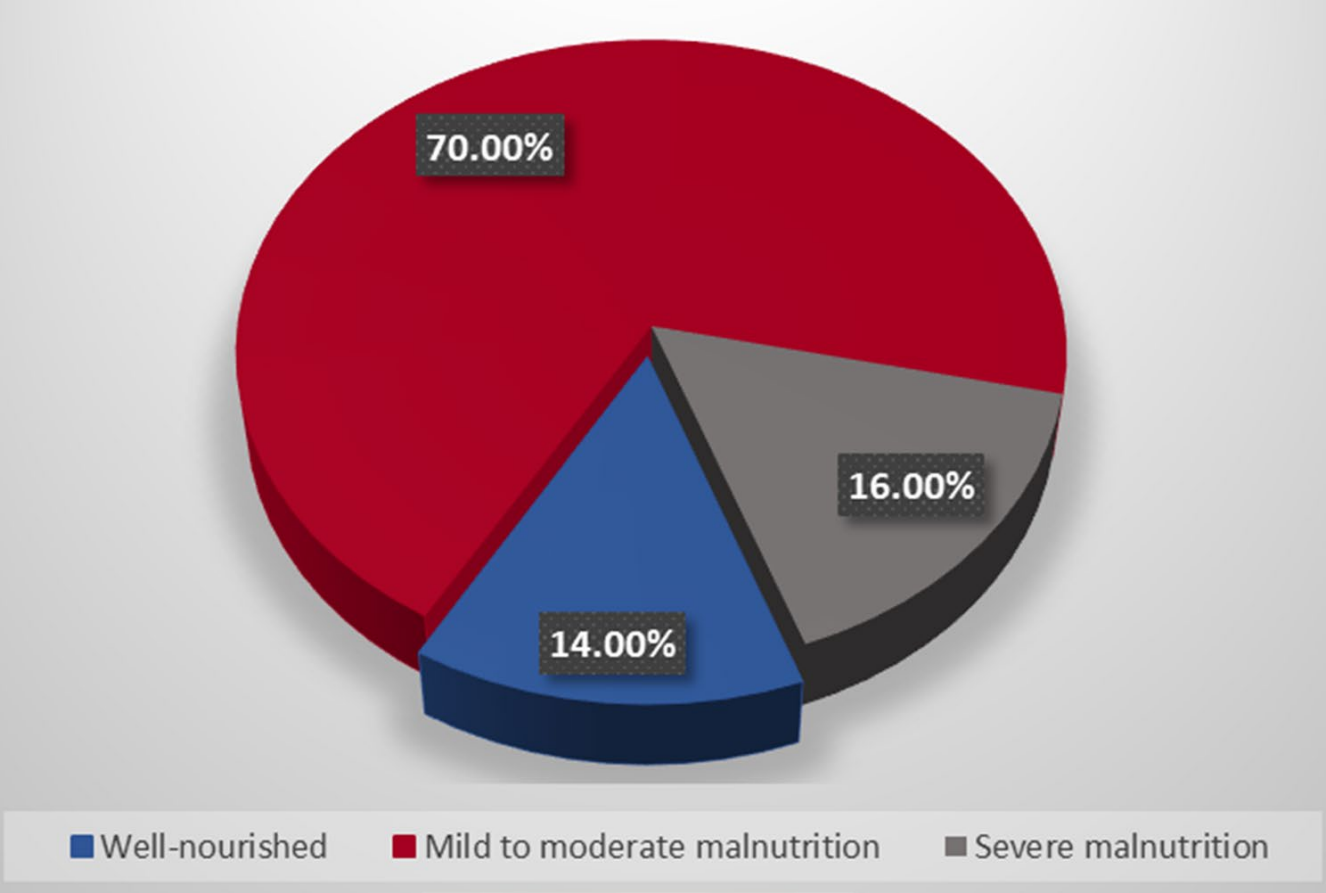
Nutritional status of the studied patients

Nutritional status was assessed utilizing the **SGA** score, with a mean ± SD of 13.54 ± 4.32. The prevalence of malnutrition was 86%; 105 (70%) patients exhibited mild to moderate malnutrition. Additionally, 24 (16%) patients demonstrated severe malnutrition while 21 (14%) patients were well-nourished, as shown in Fig. 2. **QOL** was evaluated using the **SF-12**, the mean ± SD of the PCS was 44.81 ± 1.45, the mean ± SD of the MCS was 47.38 ± 1.44 whereas the mean ± SD of the total score was 92.19 ± 2.14.

**Fig. 3:**
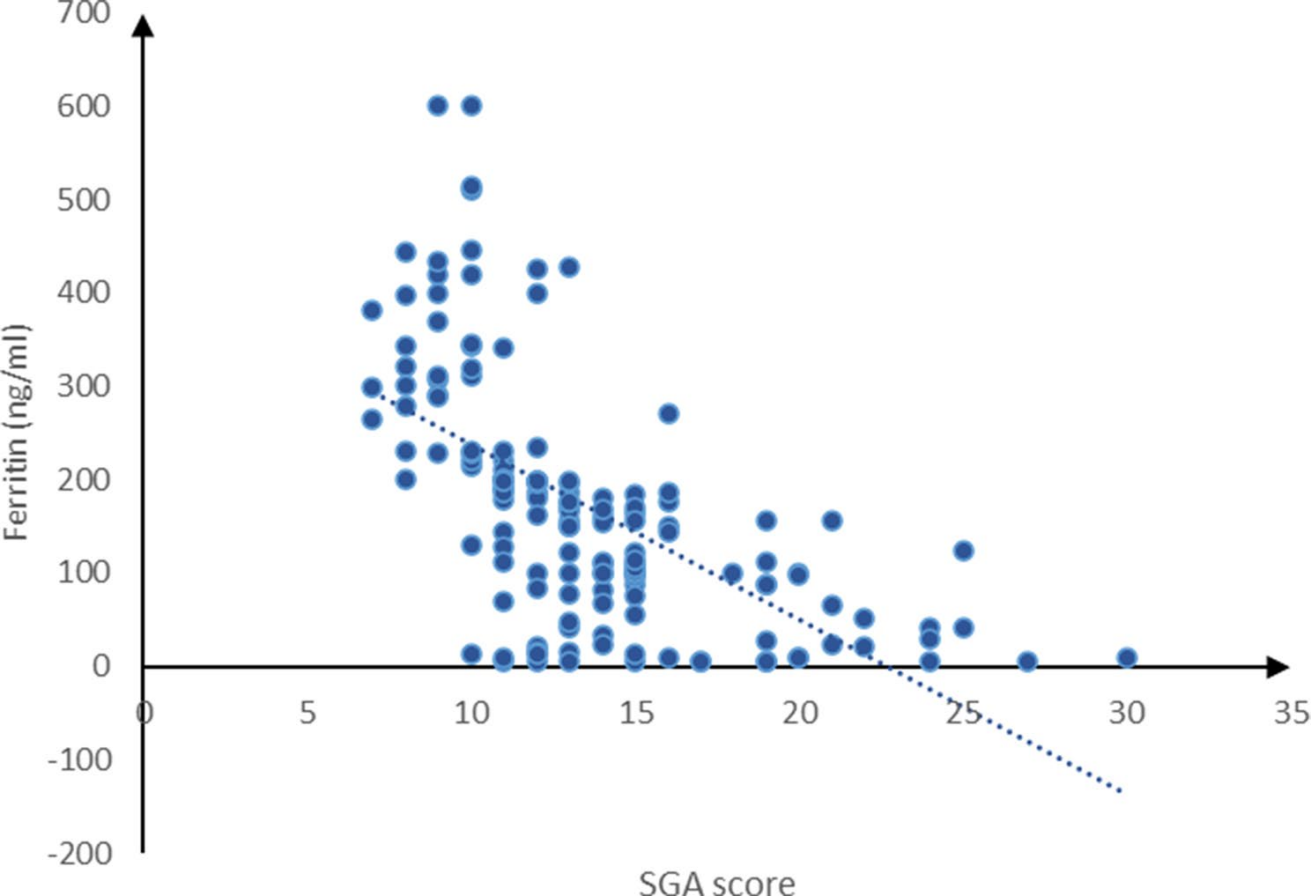
Correlation between SGA score and ferritin

**Fig. 4:**
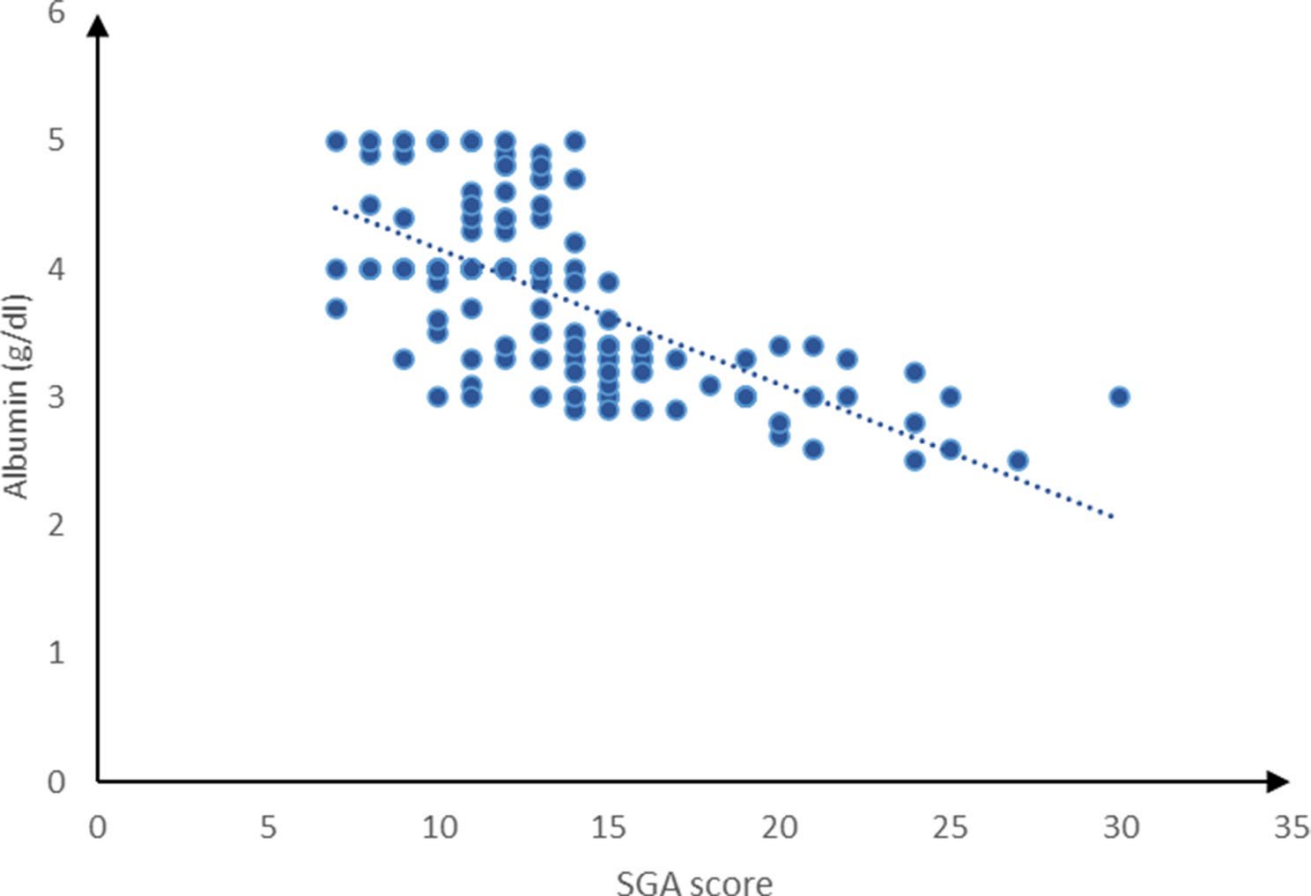
Correlation between SGA score and albumin

The current study revealed a marked negative association between SGA score and age (*P* = 0.029), BMI (*P* = 0.002), cholesterol (*P* < 0.001), TIBC (*P* < 0.001), ferritin (*P* < 0.001), TG (*P* = 0.029) and albumin (*P* < 0.001). A substantial positive correlation was detected between the SGA score and the duration of dialysis (*P* < 0.001), whereas the SGA score exhibited a non-significant correlation with Hb, as shown in Figs. 3 and 4.

Table 4 indicates that the SGA score was significantly higher in subjects with mild to moderate and severe malnutrition than well-nourished patients. There was a notable increase in those with severe malnutrition compared to those with mild to moderate malnutrition (***P* < 0.001**). BMI was markedly decreased in subjects with severe malnutrition than those with mild to moderate malnutrition (***P* = 0.004**). Additionally, dialysis duration was markedly reduced in well-nourished patients than in those with mild to moderate and severe malnutrition (***P* < 0.001**). TIBC, ferritin and albumin levels were markedly decreased in subjects with mild to moderate and severe malnutrition than well-nourished patients (***P* < 0.001**). Cholesterol and TG levels substantially declined in patients with severe malnutrition compared to well-nourished patients and those with mild to moderate malnutrition (***P* ≤ 0.001**).

Patients with mild to moderate malnutrition exhibited a significant decrease in PCS compared to well-nourished patients (***P* < 0.05**). No substantial variations were detected between well-nourished patients and those with severe malnutrition, nor between mild to moderate and severe malnutrition. Additionally, age, education level, sex, occupation, marital status, number of sessions per week, Hb, MCS and diabetic patients showed no significant differences across the different nutritional statuses.

**Table 4:** Patients-related data according to the nutritional status

		Well-nourished (*n* = 21)	Mild to moderate malnutrition (*n* = 105)	Severe malnutrition (*n* = 24)	*P* value	
Age (years)		49.9 ± 17.8	49.38 ± 13.2	44.58 ± 13.85	0.293	
Sex	**Male**	12 (57.14%)	48 (45.71%)	10 (41.67%)	0.547	
	**Female**	9 (42.86%)	57 (54.29%)	14 (58.33%)		
BMI (kg/m^2^)		21.26 ± 4.7	22.89 ± 5.42	19.12 ± 3.86	0.004*	P1 = 0.379 P2 = 0.343 P3 = 0.004*
Marital state	**Single**	3 (14.29%)	14 (13.33%)	2 (8.33%)	0.438	
	**Married**	17 (80.95%)	78 (74.29%)	18 (75%)		
	**Divorced**	1 (4.76%)	7 (6.67%)	4 (16.67%)		
	**Widow**	0 (0%)	6 (5.71%)	0 (0%)		
Occupation		4 (19.05%)	26 (24.76%)	1 (4.17%)	0.078	
Education level	**Low**	3 (23.08%)	15 (20%)	5 (38.46%)	0.524	
	**Moderate**	8 (61.54%)	39 (52%)	6 (46.15%)		
	**High**	2 (15.38%)	21 (28%)	2 (15.38%)		
Duration of dialysis (years)		2 (1–2.25)	3 (2–6)	4.5 (2–8)	< 0.001*	P1 < 0.001* P2 < 0.001* P3 = 0.417
Number of sessions per week	**1**	0 (0%)	1 (0.95%)	0 (0%)	0.401	
	**2**	8 (38.1%)	22 (20.95%)	3 (12.5%)		
	**3**	13 (61.9%)	79 (75.24%)	21 (87.5%)		
	**4**	0 (0%)	3 (2.86%)	0 (0%)		
Diabetes		2 (9.52%)	24 (22.86%)	1 (4.17%)	0.055	
Hb (g/dL)		9.83 ± 1.67	9.58 ± 1.53	9.18 ± 1.68	0.363	
TIBC (µg/dL)		369.67 ± 63.59	243.99 ± 59.22	149.71 ± 29.72	< 0.001*	P1 < 0.001* P2 < 0.001* P3 < 0.001*
Ferritin (ng/mL)		310 (288–398)	160 (96–198)	41.5 (10–98.5)	< 0.001*	P1 < 0.001* P2 < 0.001* P3 < 0.001*
Cholesterol (mg/dL)		187.05 ± 46.31	177.96 ± 46.45	135.67 ± 45.38	< 0.001*	P1 = 0.690 P2 = 0.001* P3 < 0.001*
TG (mg/dL)		179 (133–210)	147 (100–212)	78.5 (71.5–137)	< 0.001*	P1 = 0.196 P2 < 0.001* P3 < 0.001*
Albumin (g/dL)		4.32 ± 0.52	3.86 ± 0.62	2.96 ± 0.26	< 0.001*	P1 = 0.003* P2 < 0.001* P3 < 0.001*
SGA score		8.33 ± 0.73	12.75 ± 1.92	21.54 ± 3.04	< 0.001*	P1 < 0.001* P2 < 0.001* P3 < 0.001*
QOL	**PCS**	45.67 ± 1.83	44.59 ± 1.37	45 ± 1.1	0.005*	P1 = 0.005* P2 = 0.254 P3 = 0.404
	**MCS**	47.62 ± 0.97	47.35 ± 1.41	47.29 ± 1.88	0.703	
	**Total score**	93.29 ± 2.17	91.94 ± 2.06	92.29 ± 2.26	0.030*	P1 = 0.023* P2 = 0.258 P3 = 0.745

BMI: body mass index, Hb: hemoglobin, TIBC: total iron binding capacity, TG: triglycerides, SGA: subjective global assessment, QOL: quality of life, PCS: physical component summary, MCS: mental component summary

*: significant as P value ≤ 0.05, P1: P value between well-nourished and mild to moderate malnutrition, P2: P value between well-nourished and severe malnutrition, P3: P value between mild to moderate malnutrition and severe malnutrition

Among the predictors of malnutrition, TIBC (B = -0.021, P value < 0.001), ferritin (B = -0.008, P value = 0.001) and albumin (B = -1.361, P value = 0.004) were significant negative predictors of SGA score, indicating that higher values of these variables were associated with lower nutritional status scores. Other variables such as age, BMI, duration of dialysis, Hb, cholesterol and TG were not significant predictors in the model (P value > 0.05) (Table 5).

**Table 5:** Multiple linear regression analysis predicting SGA score

Predictors	B (Unstandardized)	SE	T	*P* value	95% CI Lower	95% CI Upper
Age (years)	-0.005	0.019	-0.281	0.779	-0.043	0.032
BMI (kg/m^2^)	-0.096	0.052	-1.866	0.064	-0.198	0.006
Duration of dialysis (years)	-0.058	0.094	-0.611	0.542	-0.244	0.129
Hb (g/dL)	-0.058	0.178	-0.327	0.744	-0.409	0.293
TIBC (µg/dL)	-0.021	0.004	-5.503	< 0.001*	-0.029	-0.014
Ferritin (ng/mL)	-0.008	0.002	-3.279	0.001*	-0.012	-0.003
Cholesterol (mg/dL)	-0.002	0.007	-0.250	0.803	-0.015	0.012
TG (mg/dL)	-0.001	0.003	-0.154	0.878	-0.007	0.006
Albumin (g/dL)	-1.361	0.465	-2.927	0.004*	-2.281	-0.442

SGA: subjective global assessment, BMI: body mass index, Hb: haemoglobin, TIBC: total iron binding capacity, TG: triglycerides, r: correlation coefficient, SE: standard error, CI: confidence interval, *: significant as P value ≤ 0.05

Regarding QOL, a marked negative association was identified between PCS and age (***P* < 0.001**), BMI (***P* = 0.024**) and SGA score (***P* = 0.042**) whereas no significant correlation was found with duration of dialysis or number of sessions per week. A notable negative correlation was identified between MCS and age (***P* = 0.024)**. Finally, the total QOL score was markedly diminished in diabetic patients relative to non-diabetic patients (***P* = 0.028**), no notable variations were detected concerning sex, occupation and education level.

## Discussion

Malnutrition is prevalent in ESRD patients undergoing HD. It correlates with increased morbidity, higher hospitalization rates and reduced functional capacity, all of which can lead to diminished health-related QOL. Monitoring patient’s diets and nutritional status can identify irregularities, allowing for corrections that enhance health and QOL. Therefore, evaluating patient’s nutritional status is essential [13].

This study was done for the first time in the **Middle Eastern community in Egypt**, which had specific culture and climate (very hot weather), also there is difference in the nature of diseases and response to treatment.

The present study included 150 participants with average age of 48 years and different comorbidities, most of them were females. **Mohammed et al.** [14] illustrated that HD patient’s mean age was 51.16 ± 15.03, 23% of patients were diagnosed with coronary artery disease, 68% with hypertension and 32% with diabetes. **Tayyem and Mrayyan** [15] demonstrated that HD patient’s mean age was 43.9 ± 14.6, with 94 females and 84 males among the 178 studied patients. Also, **AmirKhanlu et al.** [16] found that HD patient’s mean age in Gorgan was 55.59 ± 17.29 years, 34.48% of them had diabetes, 4.4% had hyperlipidemia, 1.8% had coronary artery disease, one case of Alzheimer’s disease and one case of Down syndrome.

Nutritional status was assessed using the SGA score that revealed a high prevalence of malnutrition among HD patients; most of them had mild to moderate malnutrition. Several studies agree with our results as: **AmirKhanlu et al.** [16] who demonstrated that 9% of subjects had severe malnutrition, 69.82% had mild to moderate malnutrition and 29.66% had normal nutritional status. **Morais et al.** [17] found that 90.9% of patients experienced mild to moderate malnutrition while 4.6% were classified as having severe malnutrition, aligning with the current findings. Conversely, the mean malnutrition score reported by **Mohammed et al.** [14] was 18.61 ± 6.17, whereas **Jahromi et al.** [18] in Iran reported a mean score of 16.6 ± 5.19, the research conducted by **Janardhan et al.** [19] in India reported a mean score of 17.9 ± 4.85 with marked negative correlation between the weight of HD patients and their nutritional status. While **Demirağ et al.** [20] found no evidence of a link between the nutritional status and BMI of HD patients.

Malnutrition is prevalent among HD patients especially those on long-term HD, **Behzad et al.** [21] demonstrated that prolonged hemodialysis correlates with increased malnutrition levels. Conversely, research by **Al-Jahdali et al.** [22] and **Alharbi et al.** [23] indicated a non-significant association between hemodialysis duration and patients’ nutritional status, which is inconsistent with our findings. The discrepancies in results may be attributed to variations in study populations, methods, locations, cultural or nutritional differences across various countries or HD centers and other factors influencing nutritional status, as education, etc.

Our results indicated that age, education level, sex, occupation, marital status, MCS, Hb levels and frequency of sessions per week exhibited non-significant differences across various nutritional statuses. **Ali et al.** [24] illustrated that measured variables such as gender, BMI, education, income, residency, marital status, occupation, dialysis sessions per week and transplantation history, did not exhibit a substantial association with malnutrition status in HD Patients. **Behzad et al.** [21] found no significance between malnutrition and the age of the patients, which is inconsistent with our results. Furthermore, **Oliveira et al.** [25] found a markedly higher prevalence of malnutrition in the elderly relative to those less than 60 years of age.

QOL was evaluated based on the SF-12 and the results illustrated decrease in the QOL in diabetic compared to non-diabetic patients with negative correlation existed between PCS & age, BMI and SGA score. **Viramontes-Hörner et al.** [26] stated that malnutrition assessed by SGA was the only factor independently and negatively associated with QOL; their findings indicated low MCS and PCS scores (< 50) in comparison to our study population. **Ohri-Vachaspati & Sehgal** [27] and **Lase’s** [28] demonstrated that insufficient protein nutrition, indicated by low serum albumin levels and low protein nitrogen appearance was independently linked to poor QOL. **Osthus et al.** [29] reported that diabetic patients undergoing dialysis experienced significantly more complications and had a poorer QOL compared to diabetic patients not undergoing dialysis. Furthermore, **Feroze et al.** [30] demonstrated a negative correlation between BMI and QOL suggesting that obese patients undergoing MHD tend to perceive a lower QOL.

## Conclusion

Malnutrition is prevalent among hemodialysis patients in Aswan, particularly in individuals receiving long-term HD. The majority of patients exhibited mild to moderate malnutrition, whereas severe malnutrition was infrequent. Malnourished patients exhibited low BMI, TIBC, albumin, cholesterol, TG and ferritin levels. Malnutrition, aging, and high BMI are independent factors linked to diminished QOL. Regular screening for malnutrition in HD patients is recommended especially for those on HD for long time, along with timely and appropriate treatment to improve the quality of life, finally it is very important to educate and encourage our patients for having healthy food to avoid malnutrition.

The current study has some limitations. Consequently, larger multi-center longitudinal studies with a substantial sample size are required to elucidate the associations between nutritional status and QOL over time.

## Data Availability

The datasets used during the current study may be made available from the corresponding author upon reasonable request.

## Abbreviations

BMI: Body mass index
CBC: Complete blood count
CKD: Chronic kidney disease
ESRD: End stage renal disease
HD: Hemodialysis
KDOQI: Kidney Disease Outcome and Quality Initiative
MCS: Mental component summery score
MHD: Maintenance hemodialysis
PCS: Physical component summery score
PEW: Protein energy wasting
QOL: Quality of life
SF-12: 12-Item Short Form Survey
SGA: Subjective Global Assessment score
TIBC: Total iron binding capacity
